# Self and professional treatment of skin and soft tissue infections among women who inject drugs: Implications for wound care provision to prevent endocarditis

**DOI:** 10.1016/j.dadr.2022.100057

**Published:** 2022-04-29

**Authors:** Kristin E. Schneider, Rebecca Hamilton White, Saba Rouhani, Catherine Tomko, Danielle Friedman Nestadt, Susan G. Sherman

**Affiliations:** Department of Health, Behavior and Society, Johns Hopkins Bloomberg School of Public Health, 624 N. Broadway, HH904F, Baltimore, MD 21205, USA

**Keywords:** Skin and soft tissue infections, Endocarditis, Wound care, Women who inject drugs

## Abstract

**Background:**

Skin and soft tissue infections (SSTI) are common among people who inject drugs and can result in severe health consequences, including infective endocarditis. Numerous barriers to accessing care often prevent people who inject drugs from seeking healthcare including past negative healthcare experiences, transportation, and shame around drug use. These barriers can lead some people who inject drugs to self-care instead of seeking formal treatment.

**Methods:**

We explored the prevalence of SSTIs and associated treatment behaviors among women who inject drugs and sell sex (*N* = 114). Women reported their drug use and SSTI histories. Those who experienced an SSTI reported if they self-treated their SSTIs and/or sought formal treatment.

**Results:**

Half (50.0%) experienced at least one SSTI in the past 6 months. SSTIs were more common among those who injected painkillers (24.6% vs 8.8%, *p* = 0.02) and who had ever been treated for endocarditis (28.1% vs 10.5%, *p* = 0.02). SSTIs were less common among those who injected multiple times per day (17.9% vs 38.6%, *p* = 0.01) and always injected with a sterile syringe (19.3% vs 42.1%, *p* = 0.01). Among those who experienced an SSTI, most (85.7%) reported self-treating, and half (52.6%) sought formal care. The emergency room was the most common source of care (73.3%).

**Conclusions:**

When experiencing SSTIs, women often opted to self-treat rather than seek formal healthcare. A lack of formal care can lead to infections progressing to serious conditions, like endocarditis. Self-treatment with non-prescribed antibiotics may further result in antibiotic-resistant infections. Low threshold, stigma free, community-based wound care programs are warranted.

## Highlights

• Half of women who inject drugs experienced a skin or soft tissue infection (SSTI)in the past 6 months.
• Most women self-treated their SSTIs (85.7%); half sought professional care (52.6%).
• When seeking professional SSTI care, women primarily used emergency rooms.

## 1 Introduction

People who inject drugs (PWID) are at increased risk of developing skin and soft tissue infections (SSTIs) due to repeated puncture of the skin (Binswanger et al., 2000; Lloyd-Smith et al., 2005; Pollini et al., 2010). If not properly treated, SSTIs can result in severe health conditions, including endocarditis, osteomyelitis, and septicemia (Centers for Disease Control Prevention, 2001; Palepu et al., 2001; Pollini et al., 2010). While SSTIs are not the only cause of these health conditions, SSTIs are the main driver of infective endocarditis and related conditions among PWID (Ebright and Pieper, 2002). Infective endocarditis is a major cause of morbidity and mortality among people PWID; estimates of 5-year endocarditis mortality range from 12–50% (Murdoch et al., 2009; Prendergast, 2006; Thuny et al., 2012). Not only can endocarditis induce severe inflammation of and damage to the heart valves, but it can also result in additional infections in the kidneys, lungs, and brain (Harvard Medical School, 2019). PWID also have worse disease outcomes, including being more likely to have open cardiac surgery, valve-related complications, and longer hospital stays (Kadri et al., 2019; Kim et al., 2016). Endocarditis incidence and related hospitalizations have nearly tripled since the early 2000s (Kadri et al., 2019; Wurcel et al., 2016). As endocarditis typically requires hospitalization or surgery to treat resulting in large healthcare expenditures (Bates et al., 2019; Coverdale et al., 2019; Fleischauer et al., 2017), the costs of untreated SSTIs are high. Thus, prevention and early treatment of injection-related wounds should be a priority for public health programs that serve PWID.

Unfortunately, PWID often do not receive the necessary care for SSTIs in a timely manner to prevent more serious health complications, for a range of reasons. For example, PWID regularly experience stigma and discrimination in health care settings, often resulting in them avoiding or delaying care (Biancarelli et al., 2019; Grau et al., 2002; Paquette et al., 2018). PWID further experience various structural barriers to healthcare, including cost, insurance status, and transportation difficulties (Miller-Lloyd et al., 2020). PWID often attempt to treat ailments, including SSTIs, themselves rather than seeking formal care (Binswanger et al., 2000; Takahashi et al., 2003). Presently, we explore the prevalence of SSTIs, self-treatment, and professional treatment among a sample of women who inject drugs and sell sex in Baltimore City, Maryland.

## 2 Methods

### 2.1 The enabling mobilization, empowerment, risk reduction, and lasting dignity (EMERALD) study

The EMERALD Study is a two group non-randomized trial testing the effect of a place-based intervention on empowerment among female sex workers in Baltimore City, Maryland. Data used in this analysis were from the 6-month follow up survey. Inclusion criteria were being age 18 years or older, identifying as a cisgender woman, and having traded sex for money, goods, or drugs three or more times in the past 3 months. Participants were recruited through targeted, street-based recruitment in sex work hotspots (September 2017–January 2019) (Allen et al., 2019). Participants provided written informed consent to enroll and completed an audio-enhanced computer-assisted self-interviewing (A-CASI) survey, covering demographic characteristics, sex work, drug use, and mental health. Participants were compensated for the 6-month follow up visit with a $40.00 prepaid Visa debit card. All study procedures were approved by the Johns Hopkins Bloomberg School of Public Health Institutional Review Board.

### 2.2 Measures

#### 2.2.1 Skin and soft tissue infections (SSTIs)

Participants were asked “In the past 6 months, how many times have you had a skin infection or abscess?” Participants responded numerically, which we then categorized into a binary indicator. Participants who were unsure of how many SSTIs they had were considered as having an SSTI.

#### 2.2.2 Treatment of SSTIs

Women who reported having SSTIs were then asked about treating these SSTIs. They selected from a list the ways they self-treated the SSTI: lanced with a needle or blade, used a warm or cold compress, bought antibiotics on the street, used leftover antibiotics, used compression, gave it time to come to a head, or used some other method. Participants then indicated if they received treatment from a healthcare provider (yes/no). Among those who received professional treatment, participants reported where they received treatment from the following list: emergency room, primary care provider, walk-in clinic, urgent care center, Baltimore City health department, needle exchange van, or some other facility. Women could report multiple sources/methods of treatment.

#### 2.2.3 Sociodemographic characteristics

Women reported their age (in years), race (coded as non-Hispanic White, non-Hispanic Black, and other), homelessness in the past 6 months (yes/no), and hunger due to a lack of food (categorized as a binary indicator of experiencing hunger at least weekly).

#### 2.2.4 Injection drug use characteristics

Women reported how frequently they injected drugs, which were recoded as a binary indicator of injecting multiple times per day versus injecting less frequently. Women also reported which drugs they injected in the past 6 months, including cocaine, speedball (simultaneous cocaine and heroin injection), heroin, painkillers, and fentanyl. Women reported if they had been injected by someone else in the past 6 months (yes/no). Finally, participants reported if they always used sterile syringed to inject (yes/no).

#### 2.2.5 History of endocarditis treatment

Participants reported if they had ever been treated for endocarditis in their lifetime (yes/no). While this measure has a different timeframe than others used in this study, we retained it in the analysis because endocarditis is a serious health consequence often developing due to an untreated or improperly treated SSTIs.

### 2.3 Analysis

The sample was restricted to women who had completed the 6 month follow up survey and reported injecting drugs in the past 6 months, yielding an analytic sample of 114 participants. We calculated the prevalence of SSTIs, then tested for differences between SSTI categories on sociodemographic and drug use characteristics using chi square tests (or in the case of age, a t-test). Given the relatively small sample, we were only able to report the prevalence of each treatment variable. Analyzes were conducted using Stata/SE v15.1 (StataCorp LLC, 2017).

## 3 Results

Participants were predominantly non-Hispanic White (72.6%) and had an average age of 36.7 (standard deviation [SD: 8.9]). Women experienced high levels of structural vulnerability (53.5% homelessness; 50.0% weekly hunger). Less than one third (28.3%) injected drugs multiple times per day. Heroin was the most commonly injected drug (98.3%), followed by fentanyl (67.0%). Painkillers were the least commonly used drug (16.7%). 42.1% had been injected by someone else in the past 6 months. 30.7% always used sterile syringes. 19.3% reported past treatment for endocarditis (Table 1).

**Table 1:** Sample Characteristics and Drug Use by SSTI Experiences, N (%).

	Total (n=114)	No SSTIs (n=57)	SSTIs (n=57)	p
**Sociodemographic Characteristics**				
Age, mean (SD)	36.7 (8.9)	37.1 (9.6)	36.2 (8.3)	0.57
Race/Ethnicity				0.95
White, non-Hispanic	82 (72.6)	41 (73.2)	41 (71.9)	
Black, non-Hispanic	22 (19.5)	11 (19.6)	11 (19.3)	
Other	9 (8.0)	4 (7.1)	5 (8.8)	
Homeless	61 (53.5)	32 (56.1)	29 (50.9)	0.57
Hunger at least weekly	57 (50.0)	27 (47.4)	30 (52.6)	0.57
**Past 6 Month Injection Drug Use**				
Injected multiple times per day	32 (28.3)	22 (38.6)	10 (17.9)	**0.01**
Specific Drug Use				
*Cocaine*	48 (42.1)	21 (36.8)	27 (47.4)	0.26
*Speedball*	54 (47.4)	24 (42.1)	30 (52.6)	0.26
*Heroin*	112 (98.3)	57 (100)	55 (96.5)	0.15
*Painkillers*	19 (16.7)	5 (8.8)	14 (24.6)	**0.02**
*Fentanyl*	75 (67.0)	36 (63.2)	39 (70.9)	0.38
Injected by someone else	48 (42.1)	21 (36.8)	27 (47.4)	0.26
Always use sterile syringes	35 (30.7)	24 (42.1)	11 (19.3)	**0.01**
**Ever treated for endocarditis**	22 (19.3)	6 (10.5)	16 (28.1)	**0.02**

*Note*. Numbers presented in each cell reflect the cell's n and column percentage, except for age where the numbers reflect the mean and standard deviation.

Half reported at least one SSTI in the past 6 months. Sociodemographic characteristics were not significantly associated with experiencing an SSTI. Injecting multiple times per day was significantly associated with fewer SSTIs than injecting less frequently (38.6% of no SSTIs vs 17.9% of STTIs, *p* = 0.01). Painkiller use was significantly associated with higher SSTI risk (8.8% of no SSTIs vs 24.6% of SSTIs, *p* = 0.02). Always injecting with a sterile syringe was also protective against SSTIs (42.1% of no SSTIs vs 19.3% of SSTIs, *p* = 0.01). Lifetime endocarditis treatment was associated with increased STTI risk (10.5% of no SSTIs vs 28.1% of SSTIs, *p* = 0.02).

Of women who experienced SSTIs, 85.7% treated the SSTI themselves (Table 2). Among those who treated the SSTIs themselves, using a warm or cold compress (58.3%), giving it time to come to a head (50.0%), and lancing with a needle or blade (41.7%) were the most common self-treatment methods. Fewer women reported seeking professional treatment (52.6%). Among those who sought professional treatment, most (73.3%) went to the emergency room for treatment. Few participants used other facilities for SSTI treatment.

**Table 2:** Past 6 Month Experiences Treating SSTIs, N (%).

Treated SSTI Herself (n=57)	48 (85.7)
Ways of Self Treating SSTI (n=48)	
Lance with needle or blade	20 (41.7)
Warm or cold compress	28 (58.3)
Buy antibiotics on the street	11 (22.9)
Leftover antibiotics	14 (29.2)
Compression	17 (35.4)
Give it time to come to a head	24 (50.0)
Other method	10 (20.8)
**Healthcare Provider Treated SSTI (n=57)**	**30 (52.6)**
Treatment Source (n=30)	
Emergency room	22 (73.3)
Primary care provider	3 (10.0)
Walk-in clinic	4 (13.3)
Urgent care center	5 (16.7)
Baltimore City Health Department	2 (6.7)
Needle exchange van	2 (6.7)
Other facility	3 (10.0)

Note. Numbers presented in each cell reflect the cell's n and percentage.

## 4 Discussion

In this study, we explored the prevalence of SSTIs and treatment behaviors among a sample of women who inject drugs and sell sex. Half of women reported at least one SSTI in the past 6 months. This prevalence is markedly higher than most estimates from other studies among PWID, which typically find between 20–35% prevalence (Binswanger et al., 2000; Centers for Disease Control Prevention, 2001; Dahlman et al., 2015; Hope et al., 2008; Lloyd-Smith et al., 2005; Phillips and Stein, 2010; Pollini et al., 2010). The high prevalence is likely due to the sample being women who sell sex, as both gender and sex work have been previously identified as risk factors for SSTIs (Dahlman et al., 2015; Hope et al., 2008; Lloyd-Smith et al., 2005; Lloyd-Smith et al., 2008; Pollini et al., 2010). This is further compounded by the high prevalence of homelessness in this sample, another known risk factor (Dwyer et al., 2009; Lloyd-Smith et al., 2005). Nearly 20% of the sample had a history of endocarditis treatment, highlighting the urgency of wound care for this population. Higher frequencies of injecting were associated with lower SSTIs risk, contrary to our expectations. One explanation for this finding may be that those participants who injected more frequently may have more experience injecting and more robust access to harm reduction supplies, allowing them to follow safer practices (i.e., wash hands prior to injection, disinfect skin prior to injection) to avoid wounds. It is also possible that individuals who inject regularly may have more consistent drug supplies which may be protective. Alternatively, women who injected more frequently may have been less likely to report minor SSTIs as they may be viewed as a “normal” part of injection drug use. Always using sterile syringes to inject drugs was associated with lower SSTI risk, highlighting the importance of syringe service programs and other sources of sterile injection equipment in preserving the health of women who inject drugs. While syringe service programs are often thought of primarily as HIV prevention resources, they are also essential for the prevention of other serious illnesses, including endocarditis resulting from injection-related SSTIs. It is important to note that other sterile injection supplies, such as cottons, cookers, and rinse water, are also essential resources for PWID to prevent SSTIs.

Among women who experienced a SSTI, self-treatment was common. While SSTI self-care can be effective in preventing disease progression, it is important to note that improper self-treatment carries risks. Using leftover antibiotics or buying antibiotics off the street were reported by a substantial proportion of women who experienced SSTIs. Inappropriate antibiotic use, which includes obtaining antibiotics from non-provider sources, using expired drugs, and failing to complete a full course of treatment, poses a considerable health risk both to individuals themselves and the broader community by contributing to antimicrobial resistance (Kunin, 1993; Naylor et al., 2018; Tenover, 2006; Zaman et al., 2017). In the US alone, the CDC has estimated that nearly 3 million antibiotic-resistant infections result in over 35,000 fatalities and $4.6 billion in healthcare costs, annually (Centers for Disease Control Prevention, 2019). PWID are at increased risk of both inappropriate antibiotic use and antibiotic-resistant infections due to the frequency of acute infections they experience, vulnerability to infection at injection sites, and underlying social and structural health risks which result in poorer access and adherence to health resources (Starrels et al., 2009). In addition to antimicrobial self-treatment, many also reported lancing the wound themselves. It is important to situate these risks within the overall structural vulnerability of the women in this study. The high prevalence of homelessness in this sample likely means that women were attempting to treat SSTIs under poor sanitary conditions.

About half of women who experienced SSTIs sought professional treatment. The emergency room was the primary source of treatment used by women in this sample. Use of the emergency room could reflect either the comparative ease of access (i.e., no appointments needed, always open) or it could reflect that women sought formal care only when the wounds had progressed to the point that they constituted emergency situations, both of which have been consistently supported by the literature (Nambiar et al., 2018; Sterk et al., 2002). Public health efforts should aim to simultaneously reduce barriers to other forms of healthcare and make earlier detection and care easier for PWID. Low threshold wound care needs to be available in a range of settings. Integrating wound care into existing services that women already access is one approach that can help treat wounds at early stages to avoid progression to more serious infections. Such services do exist to some degree, but changes are needed to increase utilization (i.e., longer hours, always having medical staff onsite). Mobile outreach services that provide wound care, as well as other health services, are warranted for structurally vulnerable populations. People experiencing homelessness, poverty, or other forms of socioeconomic marginalization often experience insurmountable barriers in accessing formal healthcare services, meaning that mobile outreach services may be their only source of care.

Stigma is a common barrier to care seeking (Meyerson et al., 2021; Miller-Lloyd et al., 2020). PWID often report stigma and previous negative healthcare experiences as reasons for delaying care for SSTIs (Harris et al., 2018). These experiences are further compounded by fears that they would experience withdrawal or would receive inadequate pain management while undergoing treatment, ultimately delaying SSTI treatment or resulting in PWID discontinuing treatment (Monteiro et al., 2020; Summers et al., 2018). It is essential that medical interventions for SSTIs provide appropriate pain and withdrawal management and compassionate, stigma-free care.

*Limitations*. First, the sample size is modest, limiting our ability to explore correlates of treatment behaviors. We were further unable to explore reasons for choosing to self-care or seek professional care in this study, like wound severity and access barriers, topics that warrant further research. We did not have information about how individuals inject drugs to understand the relative roles of subcutaneous, intramuscular, and intravenous injection may play in wound development. Our measure of endocarditis was limited, and the timeframe did not align with other measures, making it difficult to interpret associations.

Our study explored the prevalence of SSTIs and care behaviors among a sample of women who inject drugs and sell sex. The past 6-month prevalence of SSTIs was 50%, a markedly higher proportion than in other samples of PWID. Women who experienced SSTIs primarily tried to treat their wounds themselves through a variety of methods. Women who sought professional healthcare primarily did so through the emergency department, highlighting a need for lower barrier wound care in other settings to improve treatment access, prevent disease progression, and reduce the high costs of emergency department care.

## Role of funding source

Nothing to declare.

## CRediT authorship contribution statement

**Kristin E. Schneider:** Conceptualization, Writing – original draft, Writing – review & editing. **Rebecca Hamilton White:** Conceptualization, Formal analysis, Writing – review & editing. **Saba Rouhani:** Writing – review & editing. **Catherine Tomko:** Writing – review & editing, Data curation, Supervision. **Danielle Friedman Nestadt:** Writing – review & editing, Data curation, Supervision. **Susan G. Sherman:** Writing – review & editing, Data curation, Supervision.

## Declaration of Competing Interest

Dr. Sherman serves as a witness for the plaintiffs in opioid litigation. No other authors have any conflicts to disclose.
